# 1-[5-(Dimethyl­amino)-1-naphthylsulfon­yl]imidazolidine-2-thione

**DOI:** 10.1107/S1600536810029788

**Published:** 2010-07-31

**Authors:** Yong Zhang, Lei Teng

**Affiliations:** a School of Chemical and Materials Engineering, Huangshi Institute of Technology, Huangshi 435003, People’s Republic of China

## Abstract

In the title mol­ecule, C_15_H_17_N_3_O_2_S_2_, the dihedral angle between the naphthalene ring system and the imidazole ring is 89.63 (2)°. The crystal structure is stablized by weak inter­molecuar C—H⋯π and N—H⋯π inter­actions.

## Related literature

For the applications of compounds containing a 5-(dimethyl­amino)­naphthalene-1-sulfonyl group, see: Corradini *et al.* (1996 ▶, 1997 ▶); Christoforou *et al.* (2006 ▶). For a related structure, see: Zhang *et al.* (2009 ▶). For the synthetic procedure, see: Corradini *et al.* (1996 ▶).
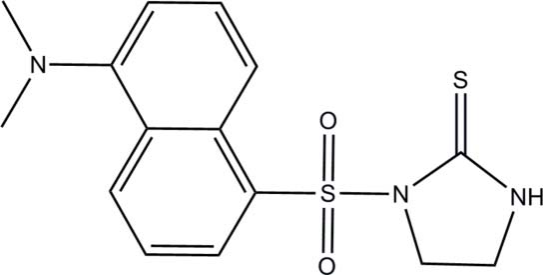


## Experimental

### 

#### Crystal data

C_15_H_17_N_3_O_2_S_2_
*M* *_r_* = 335.44
Monoclinic, 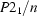

*a* = 15.364 (4) Å
*b* = 6.9814 (18) Å
*c* = 15.470 (4) Å
β = 113.967 (4)°
*V* = 1516.3 (7) Å^3^
*Z* = 4
Mo *K*α radiation
μ = 0.36 mm^−1^
*T* = 298 K
0.33 × 0.32 × 0.28 mm

#### Data collection

Bruker SMART CCD diffractometer
Absorption correction: multi-scan (*SADABS*; Sheldrick, 1997 ▶) *T*_min_ = 0.955, *T*_max_ = 0.965
8935 measured reflections
3302 independent reflections
2605 reflections with *I* > 2σ(*I*)
*R*_int_ = 0.104

#### Refinement

*R*[*F* ^2^ > 2σ(*F* ^2^)] = 0.056
*wR*(*F* ^2^) = 0.145
*S* = 1.04
3302 reflections
204 parameters
H atoms treated by a mixture of independent and constrained refinement
Δρ_max_ = 0.50 e Å^−3^
Δρ_min_ = −0.35 e Å^−3^

### 

Data collection: *SMART* (Bruker, 2007 ▶); cell refinement: *SAINT* (Bruker, 2007 ▶); data reduction: *SAINT*; program(s) used to solve structure: *SHELXS97* (Sheldrick, 2008 ▶); program(s) used to refine structure: *SHELXL97* (Sheldrick, 2008 ▶); molecular graphics: *PLATON* (Spek, 2009 ▶); software used to prepare material for publication: *SHELXTL* (Sheldrick, 2008 ▶).

## Supplementary Material

Crystal structure: contains datablocks global, I. DOI: 10.1107/S1600536810029788/lh5094sup1.cif

Structure factors: contains datablocks I. DOI: 10.1107/S1600536810029788/lh5094Isup2.hkl

Additional supplementary materials:  crystallographic information; 3D view; checkCIF report

## Figures and Tables

**Table 1:** Hydrogen-bond geometry (Å, °) *Cg*1 and *Cg*2 are the centroids of the C1–C5/C10 and C5–C10 rings, respectively.

*D*—H⋯*A*	*D*—H	H⋯*A*	*D*⋯*A*	*D*—H⋯*A*
C2—H2⋯*Cg*1^i^	0.93	2.88	3.667 (3)	143
N3—H3*A*⋯*Cg*2^ii^	0.88 (3)	2.58 (3)	3.433 (3)	165 (2)

Symmetry codes: (i) 
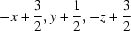
; (ii) 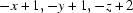.

**Fig. 1.**
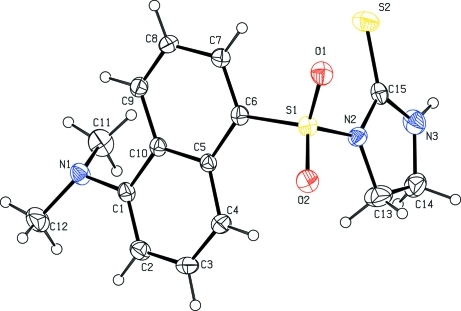
The molecular structure of (I), with displacement ellipsoids drawn at the 50% probability level.

## supplementary crystallographic information

### Comment

The dansyl (5-(dimethylamino)naphthalene-1-sulfonyl) group has been widely used as a fluorophore due to its good fluorescent properties. Recently many dansyl derivatives have been reported (Corradini *et al.*, 1996,1997; Christoforou *et al.*, 2006). We show great interest in preparing fluorescent probes that are expected to bind to hydrophobic sites in proteins or membranes and have recently published a structure reslted to the title compound (Zhang *et al.*, 2009). With this in mind, the title compound, (I), was prepared and we report herein the crystal stucture.

In the molecular structure (Fig. 1), the dihedral angle between the naphthalene ring and five-membered heterocyclic ring is 89.63 (2)°. The crystal structure is stablized by weak intermolecuar C—H···π and N—H···π interactions.

### Experimental

The intermediate *N*-(2-Aminoethyl)-5-(dimethylamino)naphthalene-1-sulfonamide was synthesized according to a literature procedure (Corradini *et al.*, 1996). Carbon bisulfide (0.76 g, 10 mmol) and sodium hydroxide(0.40 g, 10 mmol) were added into a stirred solution of the above intermediate (1.47 g, 5 mmol) in dry methanol (20 ml).The reaction mixture was allowed to stir for 24 hr at 293 K. The progress of the reaction was monitored by TLC, untill the completion of reaction. The solvent was evaporated and the residue was purified by column chromatography (dichloromethane-ethyl acetate,1:8 *v*/*v*) to afford the title compound as a yellow solid. Single crystals suitable for X-ray diffraction were prepared by slow evaporation of a solution of the title compound in dichloromethane at room temperature.

### Refinement

All H atoms were placed in idealized positions [*C*—*H*(methyl)=0.96 Å, 0.97Å (methylene) and 0.93 Å (aromatic),with *U*_iso_(H)= 1.5*U*_eq_(methyl C) 1.2*U*_eq_(other C). N-bounded hydrogen atom was found from the difference map and refined with the restraint of N—H = 0.88 (3)Å and *U*_iso_(H) = 1.2 *U*_eq_(N).

### Crystal data

**Table:**

C_15_H_17_N_3_O_2_S_2_	*F*(000) = 700
*M**_r_* = 335.44	*D*_x_ = 1.465 Mg m^−^^3^
Monoclinic, *P*2_1_/*n*	Mo *K*α radiation, λ = 0.71073 Å
Hall symbol: -P 2yn	Cell parameters from 2682 reflections
*a* = 15.364 (4) Å	θ = 2.4–28.1°
*b* = 6.9814 (18) Å	µ = 0.36 mm^−^^1^
*c* = 15.470 (4) Å	*T* = 298 K
β = 113.967 (4)°	Block, yellow
*V* = 1516.3 (7) Å^3^	0.33 × 0.32 × 0.28 mm
*Z* = 4	

### Data collection

**Table:**

Bruker SMART CCD diffractometer	3302 independent reflections
Radiation source: fine-focus sealed tube	2605 reflections with *I* > 2σ(*I*)
graphite	*R*_int_ = 0.104
φ and ω scans	θ_max_ = 27.0°, θ_min_ = 2.4°
Absorption correction: multi-scan (*SADABS*; Sheldrick, 1997)	*h* = −17→19
*T*_min_ = 0.955, *T*_max_ = 0.965	*k* = −8→8
8935 measured reflections	*l* = −19→19

### Refinement

**Table:**

Refinement on *F*^2^	Primary atom site location: structure-invariant direct methods
Least-squares matrix: full	Secondary atom site location: difference Fourier map
*R*[*F*^2^ > 2σ(*F*^2^)] = 0.056	Hydrogen site location: inferred from neighbouring sites
*wR*(*F*^2^) = 0.145	H atoms treated by a mixture of independent and constrained refinement
*S* = 1.04	*w* = 1/[σ^2^(*F*_o_^2^) + (0.071*P*)^2^] where *P* = (*F*_o_^2^ + 2*F*_c_^2^)/3
3302 reflections	(Δ/σ)_max_ = 0.001
204 parameters	Δρ_max_ = 0.50 e Å^−^^3^
0 restraints	Δρ_min_ = −0.35 e Å^−^^3^

### Special details

**Table:**

Geometry. All e.s.d.'s (except the e.s.d. in the dihedral angle between two l.s. planes) are estimated using the full covariance matrix. The cell e.s.d.'s are taken into account individually in the estimation of e.s.d.'s in distances, angles and torsion angles; correlations between e.s.d.'s in cell parameters are only used when they are defined by crystal symmetry. An approximate (isotropic) treatment of cell e.s.d.'s is used for estimating e.s.d.'s involving l.s. planes.
Refinement. Refinement of *F*^2^ against ALL reflections. The weighted *R*-factor *wR* and goodness of fit *S* are based on *F*^2^, conventional *R*-factors *R* are based on *F*, with *F* set to zero for negative *F*^2^. The threshold expression of *F*^2^ > σ(*F*^2^) is used only for calculating *R*-factors(gt) *etc*. and is not relevant to the choice of reflections for refinement. *R*-factors based on *F*^2^ are statistically about twice as large as those based on *F*, and *R*- factors based on ALL data will be even larger.

### Fractional atomic coordinates and isotropic or equivalent isotropic displacement parameters (Å^2^)

**Table:**

	*x*	*y*	*z*	*U*_iso_*/*U*_eq_	
C1	0.61885 (15)	0.6070 (3)	0.72722 (15)	0.0306 (5)	
C2	0.68621 (19)	0.7474 (3)	0.76019 (18)	0.0379 (6)	
H2	0.6868	0.8466	0.7203	0.045*	
C3	0.75453 (19)	0.7430 (3)	0.85392 (19)	0.0434 (6)	
H3	0.8011	0.8375	0.8746	0.052*	
C4	0.75429 (17)	0.6051 (3)	0.91479 (17)	0.0384 (6)	
H4	0.7983	0.6099	0.9774	0.046*	
C5	0.68768 (15)	0.4529 (3)	0.88431 (15)	0.0271 (5)	
C6	0.68432 (16)	0.2951 (3)	0.94243 (16)	0.0299 (5)	
C7	0.62594 (17)	0.1403 (3)	0.90561 (16)	0.0342 (5)	
H7	0.6253	0.0398	0.9448	0.041*	
C8	0.56701 (17)	0.1343 (3)	0.80839 (17)	0.0370 (6)	
H8	0.5298	0.0267	0.7828	0.044*	
C9	0.56426 (17)	0.2841 (3)	0.75203 (17)	0.0336 (5)	
H9	0.5235	0.2792	0.6883	0.040*	
C10	0.62191 (14)	0.4484 (3)	0.78765 (15)	0.0271 (5)	
C11	0.45077 (18)	0.6337 (5)	0.6290 (2)	0.0536 (7)	
H11A	0.4425	0.5578	0.6769	0.080*	
H11B	0.4051	0.5953	0.5678	0.080*	
H11C	0.4416	0.7665	0.6391	0.080*	
C12	0.5635 (2)	0.7304 (4)	0.5667 (2)	0.0576 (8)	
H12A	0.5604	0.8616	0.5837	0.086*	
H12B	0.5157	0.7072	0.5043	0.086*	
H12C	0.6253	0.7046	0.5677	0.086*	
C13	0.7299 (2)	0.6395 (4)	1.1372 (2)	0.0589 (8)	
H13A	0.7391	0.7055	1.0863	0.071*	
H13B	0.7896	0.6394	1.1929	0.071*	
C14	0.6522 (2)	0.7308 (4)	1.1575 (2)	0.0515 (7)	
H14A	0.6764	0.7806	1.2216	0.062*	
H14B	0.6223	0.8341	1.1135	0.062*	
C15	0.60716 (16)	0.4138 (3)	1.11357 (15)	0.0336 (5)	
N1	0.54715 (14)	0.6055 (3)	0.63401 (13)	0.0381 (5)	
N2	0.69536 (13)	0.4434 (3)	1.10932 (13)	0.0328 (4)	
N3	0.58699 (18)	0.5772 (3)	1.14554 (17)	0.0496 (6)	
H3A	0.534 (2)	0.590 (4)	1.154 (2)	0.059*	
O1	0.74468 (12)	0.1037 (2)	1.09977 (12)	0.0422 (4)	
O2	0.84230 (11)	0.3748 (3)	1.09114 (11)	0.0414 (4)	
S1	0.75069 (4)	0.29031 (8)	1.06638 (4)	0.03206 (19)	
S2	0.53971 (5)	0.22082 (10)	1.08475 (6)	0.0524 (2)	

### Atomic displacement parameters (Å^2^)

**Table:**

	*U*^11^	*U*^22^	*U*^33^	*U*^12^	*U*^13^	*U*^23^
C1	0.0297 (12)	0.0365 (13)	0.0286 (12)	0.0012 (10)	0.0150 (9)	0.0006 (9)
C2	0.0425 (14)	0.0352 (13)	0.0384 (13)	−0.0015 (10)	0.0190 (11)	0.0049 (10)
C3	0.0452 (16)	0.0396 (14)	0.0437 (15)	−0.0167 (11)	0.0165 (12)	−0.0017 (11)
C4	0.0372 (14)	0.0423 (14)	0.0316 (12)	−0.0092 (11)	0.0097 (10)	−0.0028 (10)
C5	0.0263 (11)	0.0309 (11)	0.0263 (11)	0.0000 (9)	0.0129 (8)	−0.0013 (9)
C6	0.0266 (11)	0.0343 (12)	0.0291 (11)	0.0026 (9)	0.0118 (9)	0.0001 (9)
C7	0.0402 (13)	0.0308 (12)	0.0339 (12)	−0.0022 (10)	0.0173 (10)	−0.0006 (10)
C8	0.0413 (14)	0.0339 (13)	0.0393 (13)	−0.0111 (11)	0.0200 (11)	−0.0103 (10)
C9	0.0303 (12)	0.0416 (13)	0.0295 (12)	−0.0041 (10)	0.0126 (9)	−0.0060 (10)
C10	0.0232 (10)	0.0328 (12)	0.0280 (11)	0.0021 (9)	0.0130 (9)	−0.0015 (9)
C11	0.0345 (15)	0.072 (2)	0.0477 (16)	0.0080 (14)	0.0092 (12)	0.0107 (14)
C12	0.0583 (19)	0.073 (2)	0.0356 (15)	−0.0115 (15)	0.0130 (13)	0.0146 (13)
C13	0.0640 (19)	0.0383 (15)	0.083 (2)	−0.0096 (14)	0.0386 (17)	−0.0153 (15)
C14	0.070 (2)	0.0387 (15)	0.0469 (16)	0.0002 (13)	0.0246 (15)	−0.0069 (12)
C15	0.0395 (13)	0.0371 (13)	0.0266 (11)	0.0071 (10)	0.0158 (10)	0.0067 (10)
N1	0.0322 (11)	0.0490 (12)	0.0302 (10)	0.0000 (9)	0.0099 (8)	0.0070 (9)
N2	0.0342 (11)	0.0307 (10)	0.0343 (11)	−0.0012 (8)	0.0145 (8)	−0.0036 (8)
N3	0.0595 (15)	0.0440 (13)	0.0595 (15)	0.0068 (11)	0.0389 (13)	−0.0025 (11)
O1	0.0509 (11)	0.0343 (9)	0.0399 (10)	0.0105 (8)	0.0169 (8)	0.0090 (7)
O2	0.0284 (9)	0.0521 (11)	0.0391 (10)	0.0026 (8)	0.0092 (7)	0.0035 (8)
S1	0.0314 (3)	0.0339 (3)	0.0303 (3)	0.0052 (2)	0.0119 (2)	0.0030 (2)
S2	0.0465 (4)	0.0484 (4)	0.0687 (5)	−0.0096 (3)	0.0300 (4)	0.0000 (3)

### Geometric parameters (Å, °)

**Table:**

C1—C2	1.365 (3)	C11—H11B	0.9600
C1—N1	1.415 (3)	C11—H11C	0.9600
C1—C10	1.437 (3)	C12—N1	1.456 (3)
C2—C3	1.403 (4)	C12—H12A	0.9600
C2—H2	0.9300	C12—H12B	0.9600
C3—C4	1.348 (3)	C12—H12C	0.9600
C3—H3	0.9300	C13—N2	1.468 (3)
C4—C5	1.417 (3)	C13—C14	1.496 (4)
C4—H4	0.9300	C13—H13A	0.9700
C5—C10	1.425 (3)	C13—H13B	0.9700
C5—C6	1.436 (3)	C14—N3	1.428 (3)
C6—C7	1.372 (3)	C14—H14A	0.9700
C6—S1	1.769 (2)	C14—H14B	0.9700
C7—C8	1.406 (3)	C15—N3	1.329 (3)
C7—H7	0.9300	C15—N2	1.398 (3)
C8—C9	1.351 (3)	C15—S2	1.647 (2)
C8—H8	0.9300	N2—S1	1.6628 (19)
C9—C10	1.417 (3)	N3—H3A	0.88 (3)
C9—H9	0.9300	O1—S1	1.4182 (17)
C11—N1	1.464 (3)	O2—S1	1.4275 (17)
C11—H11A	0.9600		
			
C2—C1—N1	123.0 (2)	N1—C12—H12A	109.5
C2—C1—C10	119.4 (2)	N1—C12—H12B	109.5
N1—C1—C10	117.6 (2)	H12A—C12—H12B	109.5
C1—C2—C3	120.4 (2)	N1—C12—H12C	109.5
C1—C2—H2	119.8	H12A—C12—H12C	109.5
C3—C2—H2	119.8	H12B—C12—H12C	109.5
C4—C3—C2	121.7 (2)	N2—C13—C14	103.6 (2)
C4—C3—H3	119.2	N2—C13—H13A	111.0
C2—C3—H3	119.2	C14—C13—H13A	111.0
C3—C4—C5	120.7 (2)	N2—C13—H13B	111.0
C3—C4—H4	119.7	C14—C13—H13B	111.0
C5—C4—H4	119.7	H13A—C13—H13B	109.0
C4—C5—C10	118.31 (19)	N3—C14—C13	103.3 (2)
C4—C5—C6	124.9 (2)	N3—C14—H14A	111.1
C10—C5—C6	116.72 (19)	C13—C14—H14A	111.1
C7—C6—C5	121.9 (2)	N3—C14—H14B	111.1
C7—C6—S1	115.27 (17)	C13—C14—H14B	111.1
C5—C6—S1	122.80 (17)	H14A—C14—H14B	109.1
C6—C7—C8	119.7 (2)	N3—C15—N2	105.5 (2)
C6—C7—H7	120.1	N3—C15—S2	125.9 (2)
C8—C7—H7	120.1	N2—C15—S2	128.54 (17)
C9—C8—C7	120.3 (2)	C1—N1—C12	115.8 (2)
C9—C8—H8	119.8	C1—N1—C11	113.87 (19)
C7—C8—H8	119.8	C12—N1—C11	110.3 (2)
C8—C9—C10	121.7 (2)	C15—N2—C13	111.46 (19)
C8—C9—H9	119.2	C15—N2—S1	125.90 (16)
C10—C9—H9	119.2	C13—N2—S1	122.26 (17)
C9—C10—C5	119.38 (19)	C15—N3—C14	115.8 (2)
C9—C10—C1	121.37 (19)	C15—N3—H3A	121 (2)
C5—C10—C1	119.17 (19)	C14—N3—H3A	123.1 (19)
N1—C11—H11A	109.5	O1—S1—O2	119.00 (10)
N1—C11—H11B	109.5	O1—S1—N2	108.99 (10)
H11A—C11—H11B	109.5	O2—S1—N2	103.62 (10)
N1—C11—H11C	109.5	O1—S1—C6	108.55 (11)
H11A—C11—H11C	109.5	O2—S1—C6	110.75 (10)
H11B—C11—H11C	109.5	N2—S1—C6	104.94 (10)
			
N1—C1—C2—C3	178.9 (2)	C2—C1—N1—C12	16.4 (3)
C10—C1—C2—C3	−3.7 (4)	C10—C1—N1—C12	−161.2 (2)
C1—C2—C3—C4	−2.1 (4)	C2—C1—N1—C11	−113.0 (3)
C2—C3—C4—C5	3.6 (4)	C10—C1—N1—C11	69.5 (3)
C3—C4—C5—C10	0.7 (3)	N3—C15—N2—C13	−2.2 (3)
C3—C4—C5—C6	177.1 (2)	S2—C15—N2—C13	177.2 (2)
C4—C5—C6—C7	−171.7 (2)	N3—C15—N2—S1	−175.13 (17)
C10—C5—C6—C7	4.8 (3)	S2—C15—N2—S1	4.3 (3)
C4—C5—C6—S1	11.1 (3)	C14—C13—N2—C15	−1.1 (3)
C10—C5—C6—S1	−172.47 (15)	C14—C13—N2—S1	172.14 (18)
C5—C6—C7—C8	−0.3 (3)	N2—C15—N3—C14	5.0 (3)
S1—C6—C7—C8	177.21 (17)	S2—C15—N3—C14	−174.4 (2)
C6—C7—C8—C9	−3.2 (3)	C13—C14—N3—C15	−5.7 (3)
C7—C8—C9—C10	1.9 (4)	C15—N2—S1—O1	−44.0 (2)
C8—C9—C10—C5	2.9 (3)	C13—N2—S1—O1	143.8 (2)
C8—C9—C10—C1	179.8 (2)	C15—N2—S1—O2	−171.64 (17)
C4—C5—C10—C9	170.7 (2)	C13—N2—S1—O2	16.1 (2)
C6—C5—C10—C9	−6.0 (3)	C15—N2—S1—C6	72.1 (2)
C4—C5—C10—C1	−6.3 (3)	C13—N2—S1—C6	−100.1 (2)
C6—C5—C10—C1	177.01 (18)	C7—C6—S1—O1	12.6 (2)
C2—C1—C10—C9	−169.1 (2)	C5—C6—S1—O1	−169.92 (17)
N1—C1—C10—C9	8.5 (3)	C7—C6—S1—O2	145.02 (18)
C2—C1—C10—C5	7.8 (3)	C5—C6—S1—O2	−37.5 (2)
N1—C1—C10—C5	−174.59 (19)	C7—C6—S1—N2	−103.77 (18)
N2—C13—C14—N3	3.7 (3)	C5—C6—S1—N2	73.67 (19)

### Hydrogen-bond geometry (Å, °)

**Table:**

Cg1 and Cg2 are the centroids of the C1–C5/C10 and C5–C10 rings, respectively.

**Table:**

*D*—H···*A*	*D*—H	H···*A*	*D*···*A*	*D*—H···*A*
C2—H2···Cg1^i^	0.93	2.88	3.667 (3)	143
N3—H3A···Cg2^ii^	0.88 (3)	2.58 (3)	3.433 (3)	165 (2)

Symmetry codes: (i) −*x*+3/2, *y*+1/2, −*z*+3/2; (ii) −*x*+1, −*y*+1, −*z*+2.

## References

[bb1] Bruker (2007). *SAINT-Plus* and *SMART* Bruker AXS Inc., Madison, Wisconsin, USA.

[bb2] Christoforou, A. M., Marzilli, P. A. & Marzilli, L. G. (2006). *Inorg. Chem.* **45**, 6771–6781. 10.1021/ic0606375 16903734

[bb3] Corradini, R., Dossena, A., Galaverna, G., Marchelli, R., Panagia, A. & Sarto, G. (1997). *J. Org. Chem.* **62**, 6283–6289.

[bb4] Corradini, R., Dossena, A., Marchelli, R., Panagia, A., Sartor, G., Saviano, M., Lombardi, A. & Pavone, V. (1996). *Chem. Eur. J.* **2**, 373–381.

[bb5] Sheldrick, G. M. (1997). *SADABS* University of Göttingen, Germany.

[bb6] Sheldrick, G. M. (2008). *Acta Cryst.* A**64**, 112–122. 10.1107/S0108767307043930 18156677

[bb7] Spek, A. L. (2009). *Acta Cryst.* D**65**, 148–155. 10.1107/S090744490804362X PMC2631630 19171970

[bb8] Zhang, Y., Qu, Y. & Liu, T. (2009). *Acta Cryst.* E**65**, o2752. 10.1107/S1600536809041476 PMC2971309 21578346

